# Implementing and evaluating a high‐resolution diode array for patient‐specific quality assurance of robotic brain stereotactic radiosurgery/radiotherapy

**DOI:** 10.1002/acm2.13569

**Published:** 2022-03-12

**Authors:** Qianyi Xu, Kiet Huynh, Wei Nie, Mark S. Rose, Ashish K. Chawla, Kevin S. Choe, Samir Kanani, Gregory J. Kubicek, Jiajin Fan

**Affiliations:** ^1^ Department of Advanced Radiation Oncology and Proton Therapy Inova Schar Cancer Institute Fairfax Virginia USA; ^2^ Sun Nuclear Corporation Melbourne Florida USA; ^3^ Department of Radiation Oncology MD Anderson Cancer Center at Cooper Camden New Jersey USA

**Keywords:** CyberKnife, diode array, PSQA

## Abstract

The purpose of the study was to introduce and evaluate a high‐resolution diode array for patient‐specific quality assurance (PSQA) of CyberKnife brain stereotactic radiosurgery (SRS) and stereotactic radiotherapy (SRT). Thirty‐three intracranial plans were retrospectively delivered on the SRS MapCHECK using fixed cone, Iris, and multileaf collimator (MLC). The plans were selected to cover a range of sites from large tumor bed, single/multiple small brain metastases (METs) to trigeminal neuralgia. Fiducial tracking using the four fiducials embedded around the detector plane was used as image guidance. Results were analyzed before and after registration based on absolute dose gamma criterion of 1 mm distance‐to‐agreement and 0.5%–3% dose‐difference. Overall, the gamma passing rates (1 mm and 3% criterion) before registration for all the patients were above 90% for all three treatment modalities (96.8 ± 3.5%, the lowest passing rate of 90.4%), and were improved after registration (99.3 ± 1.5%). When tighter criteria (1 mm and 2%) were applied, the gamma passing rates after registration for all the cases dropped to 97.3 ± 3.2%. For trigeminal neuralgia cases, we applied 1 mm and 0.5% criterion and the passing rates dropped from 100 ± 0.0% to 98.5 ± 2.0%. The mean delivery time was 33.4 ± 11.7 min, 24.0 ± 4.9 min, and 17.1 ± 2.6 min for the fixed cone, Iris, and MLC, respectively. With superior gamma passing rates and reasonable quality assurance (QA) time, we believe the SRS MapCHECK could be a good option for routine PSQA for CyberKnife SRS/SRT.

## 1 INTRODUCTION

Stereotactic radiosurgery/radiotherapy (SRS/SRT) delivers very conformal and high gradient dose to lesions in the brain. Great challenges have been posed for patient‐specific quality assurance (PSQA) due to lack of efficient and reliable methods. Conventional farmer‐type ion chambers are not suitable for small‐field measurement due to volume averaging effects. Smaller ion chambers with sensitivity volume approximately 0.01 cm^3^ are more appropriate for SRS/SRT PSQA. However, large output variations exceeding 15% were observed when measuring field size down to 5 mm × 5 mm.^1^ Another commonly used method is film‐based PSQA due to its high spatial resolution. It is well known that the method is labor intensive, has a delayed readout, and is highly dependent on user processing skills.^2^ An Electronic Portal Imaging Device (EPID) is another option for PSQA due to its availability and simplicity. It is reported an EPID‐based method overly responds to low‐energy photons and dose rate dependent.^3^ Since an EPID is stationary relative to the gantry, it cannot account for any couch rotational uncertainties.

In recent years, multiple commercial PSQA devices have been introduced utilizing high‐spatial‐resolution detector arrays.^4^, ^5^, ^6^, ^7^, ^8^, ^9^, ^10^ McCulloch et al. introduced the Octavius 4D Modular Phantom with the 1000 SRS array (PTW, Freiburg, Germany) for brain SRS on the Novalis TX linac. The SRS array consists of 977 liquid‐filled ion chambers with a detector spacing of 2.5 mm in the center area (5.5 cm × 5.5 cm). Xia et al.^11^ reported PSQA results of 275 SRS/stereotactic body radiation therapy (SBRT) plans on linac using the Delta4 device (ScandiDos AB, Uppsala, Sweden) and SRS MapCHECK (Sun Nuclear, Melbourne, FL, USA). Both devices are suitable for SRS PSQA due to their high‐spatial‐resolution detector. Delta4 has 1069 p‐type diodes installed on the two near‐orthogonal panels with spatial resolution of 5 mm in the inner area. SRS MapCHECK has 1013 n‐type solid‐state diodes in the 77 × 77 mm^2^ measurement areas. Valve et al.^12^ reported PSQA results for stereotactic volumetric modulated arc therapy (VMAT) treatment plans using a linac‐head mounted ionization chamber array (MatriXX, IBA, Schwarzenbruck, Germany). The array consisted of 1020 air‐vented ionization chambers with 7.62 mm spatial resolution. In all these reports, promising PSQA results were achieved from the high‐spatial‐resolution detector arrays.

Historically, PSQA for CyberKnife SRS/SRT has been difficult due to lack of equipment for effective and efficient measurement and relatively long delivery time, and relevant reports were very rare. Koksal et al.^13^ performed PSQA for 25 CyberKnife SRS patients using a PinPoint ionization chamber. Milder et al.^14^ reported 84 PSQA for CyberKnife multileaf collimator (MLC) plans using an Octavius SRS 1000 array (PTW). Vandervoort et al.^15^ performed CyberKnife PSQA for nine patients using radiochromic film and an A16 small volume chamber in a head phantom. Other novel detector arrays have been proposed and hold promise for PSQA.^16^, ^17^ Recently CyberKnife delivery efficiency has been greatly improved with the newly introduced Volo optimization engine.^18^, ^19^, ^20^ The total number of monitor unit (MU) and beams have been greatly reduced compared with plans using the prior sequential optimizer. With the availability of high‐spatial‐resolution diode arrays, PSQA for CyberKnife SRS may be feasible as part of clinical workflow. In this study, we will first introduce a step‐by‐step process on how to implement the SRS MapCHECK for CyberKnife SRS/SRT PSQA. Second, we will report our SRS/SRT PSQA results based on clinical sites from large tumor bed, single/multiple small brain metastases (METs) to trigeminal neuralgia (TGN). We will also compare delivery time and accuracy between different delivery modalities from fixed cone, Iris to MLC.

## 2 MATERIALS AND METHODS

### 2.1 SRS MapCHECK and StereoPHAN phantom

The use of the SRS MapCHECK on the linac platform was introduced by Rose et al.^6^ The detector array consisted of 1013 n‐type solid‐state diodes, which were a variant of EDGE detector (Sun Nuclear). For each diode, the measurement area was 0.48 × 0.48 mm^2^, measurement volume was 0.007 mm^3^, and spatial resolution was 2.47 mm. The detector array was sandwiched by two pieces of rectangular polymethyl methacrylate (PMMA) blocks. Four fiducials were embedded around the detector array and can be used for CyberKnife fiducial tracking. The detector array and two blocks were housed inside the Sun Nuclear's end‐to‐end phantom, the StereoPHAN, during delivery. The StereoPHAN could be locked on the couch and the initial setup could be done by aligning lasers to the marked lines on the StereoPHAN. SNC Patient software is used for operation of the SRS MapCHECK. CyberKnife PSQA is only supported in SNC Patient software version 8.3 or later.

### 2.2 Implementing workflow

#### 2.2.1 Phantom scan and contouring

The StereoPHAN phantom with the inserted SRS MapCHECK was first scanned on a GE scanner (Discovery, GE healthcare, Chicago, IL, USA), following our CyberKnife brain scanning protocol. Three structures had to be contoured on the computed tomography (CT): the center detector, MLC target, and external body. The first two were used for dose calibration and the last one was used for density override (1.2 g/cm^3^ was used per Sun Nuclear manual). The contour set could be transferred to the scanned CT from a contouring aid dataset provided by Sun Nuclear after image registration. In our application, a third‐party software (Velocity, the Varian Medical Systems, Palo Alto, CA, USA) was used for rigid image registration.

#### 2.2.2 Array and dose calibration

**FIGURE 1:**
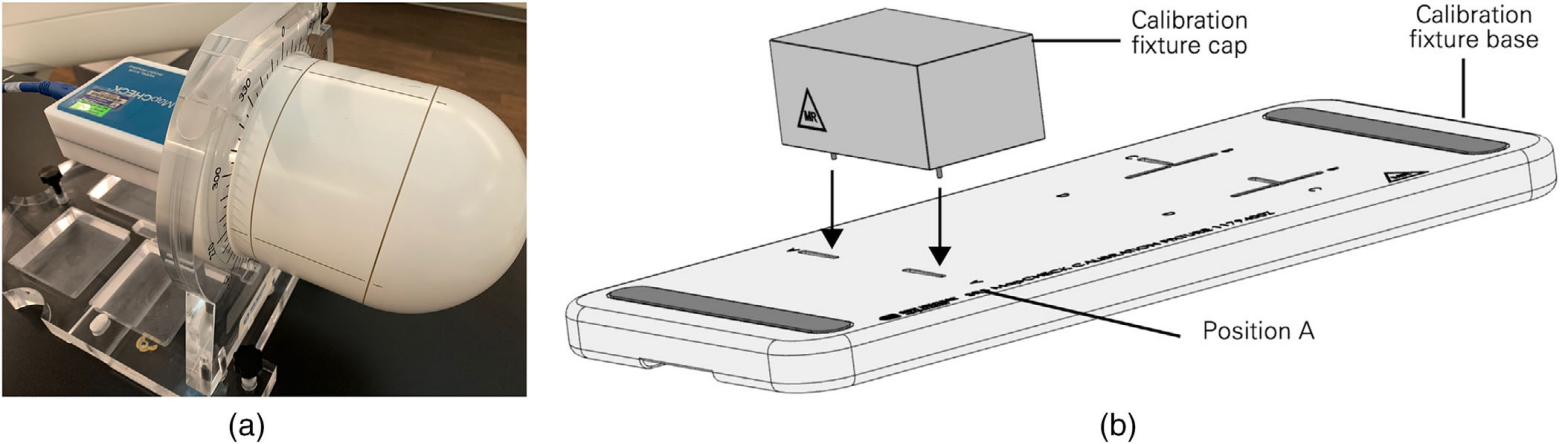
The StereoPHAN and fixture for calibration. The SteroPHAN is the cylindrical housing for the stereotactic radiosurgery (SRS) MapCHECK with two pieces of buildup spacers (a). The fixture (b) is for array calibration by irradiating the SRS MapCHECK at four marked locations from A to D

There were two calibration measurements needed before performing PSQA: array and dose calibration. The array calibration consisted of two series of steps with different phantom setups: the first one with the calibration fixture (Figure 1b) and the second one with StereoPHAN (Figure 1a). For the first measurement series with the calibration fixture, four measurements were acquired at locations of A, B, C, and D in the anterior–posterior (AP) direction and another four measurements were acquired in the posterior–anterior (PA) direction (flipping the SRS MapCHECK). It is recommended that the accuracy of all shifts be submillimeter. The fixture was installed on a couch index bar to avoid potential movement during setup. We performed the first series of array calibration steps by aligning the SRS MapCHECK with the room and linac laser for each of the eight measurements and found the resultant array calibration compromised due to positioning uncertainties. Because the array calibration is not very sensitive to the energy distribution (flattening filter free (FFF) vs. flattening filter (FF)), we used a Sun Nuclear supplied array calibration (performed at 6 MV with FF). Alternatively, Sun Nuclear recommends user perform the array calibration on a C‐arm linac using a 6 MV beam with an FF. It is worth noting that the initially released array calibration fixture was designed for C‐arm linac array calibration only and a manufacturer upgrade was needed to allow 180° device rotation and PA beam could be delivered on CyberKnife systems. The second series of steps of array calibration was to deliver 200 MU centered on the detector plane in the anterior posterior (AP) and posterior anterior (PA) directions with the SRS MapCHECK inserted in the StereoPHAN. Instead of using the room lasers for alignment, we created a phantom plan with recommended beam geometry; this removed the inherent setup error due to laser positioning accuracy allowing the user to instead setup based on the internal fiducials. The only parameter we could not achieve in the plan was source to skin distance (SSD) due to the fixed setting of source location in the single beam quality assurance (QA) plan. To achieve desired SSD, the following workaround was used. We started beam alignment based on the fiducials in the single beam QA plan. Once the beam was aligned by the default QA SSD, we paused the treatment and click the "exit" button to quit the treatment. We switched to Physics mode and manually adjusted SSD using the teach pendent to achieve desired SSD. After delivering 200 MU in the AP direction, we flipped the SRS MapCHECK and delivered another 200 MU to finish the array calibration process. After the second series of steps, the relative sensitivity of all detectors was established and the information required for diode angular correction obtained. Per Sun Nuclear recommendation, the same array calibration could be shared between fixed cones, Iris, and MLC deliveries.

Dose calibration was similar to the StereoPHAN factor measurement in the second series of steps of array calibration. The differences were (1) dose to the center detector was needed and (2) the dose calibration was needed for each delivery modality (fixed cone, Iris, and MLC). For fixed cone and Iris collimator, an isocentric plan with a single QA beam was generated with 60 mm fixed cone and Iris collimator. For MLC, a sequential optimization plan was generated with a single QA beam conforming to the central MLC target (54 × 54 mm^2^). All plans were prescribed to the mean dose of 100 cGy to the central detector in Precision. Other calibration factors are applied by Sun Nuclear, including angular correction factors, dose rate correction factors, field size correction factors, and temperature correction factors. The effects of these factors on measurement results were well studied.^21^, ^22^, ^23^ It is reported that in axial angular direction, the correction factors were <2% for most beam angles and in azimuthal direction, correction factors were <1% for 6 MV and 6 MV FFF beams energies. Ahmed et al. also found dose rate correction factors were <1% for 6 MV and <0.5% for 6 MV FFF for the evaluated dose rate variations. The field size correction factors were <2% except for 6 MV 5 × 5 mm^2^ field (3.2%). In order to compensate for the machine output variation, a TG‐51‐based monthly QA was performed before calibration and a dose correction factor was applied in the dose calibration.

#### 2.2.3 QA plan and delivery

The QA plans were first generated by overlaying clinical plans with a phantom plan. If the target was away from the alignment center in the clinical plan, an adjustment of the alignment center in the QA plan was needed to ensure all four fiducials were visible in the central part of the X‐ray images. Additionally, we needed to ensure the detector plane was in the middle portion of 3D patient dose. The deliverable plan was calculated with finite size pencil beam algorithm with high resolution. The phantom setup on the couch was quick and a rough alignment of the phantom to the room lasers were sufficient since the fiducial tracking method could fine tune phantom setup in seconds. We recommend the phantom to be placed at the most superior end of the couch to avoid potential collision warnings during delivery. After PSQA delivery, the measured dose was displayed immediately on the screen. When importing the dose from Precision, an xml file with the alignment center of the delivered QA plan was needed to register the measured and imported doses by the SNC Patient software. The user interface of the SNC Patient software was very user friendly and similar to those of ArcCheck and MapCHECK.

## 3 RESULTS

A total of 33 SRS/SRT cases were delivered on the SRS MapCHECK phantom using the CyberKnife M6 system. Fourteen cases were delivered by fixed cones, 11 cases were delivered by Iris, and eight cases were delivered by MLC. Since the fixed cones are only used for small METs in our institution, 5 mm cone was used for all the cone cases. Among all the cone cases, four cases were single small MET, five cases were multiple METs (two to five METs), and the last five cases were TGN. For multiple METs cases, it is difficult to find clinical cases that had all the METs in the same detector plane. SNC Patient software has added a QA Setup Tool that allows the user to visualize oblique planes to assist in finding the ideal plane to measure QA. To evaluate measurement accuracy, we simulated cases by adding METs in the same detector plane and planning these simulated METs following our clinical guideline. We retrospectively performed PSQA on five TGN cases as it had been extremely difficult to perform absolute dose QA for such cases. The size (the maximum size in one dimension) of the METs for the cone cases was 7.5 ± 3.4 mm (mean ± standard deviation). The size of the brain tumors for Iris and MLC cases was 24.7 ± 13.2 mm and 34.0 ± 10.5 mm, respectively. Most of the Iris and MLC cases were SRT due to faster treatment delivery for these two modalities. The mean size of the Iris collimators used for treatment was 8.9 ± 2.6 mm.

**FIGURE 2:**
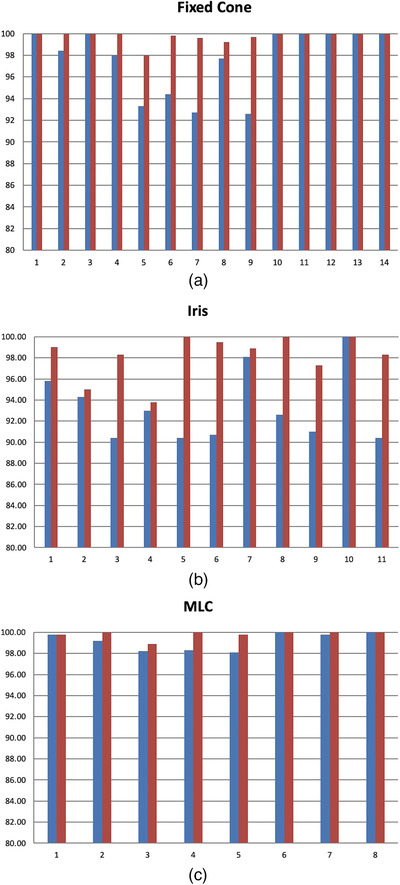
The gamma passing rates for fixed cone (a), Iris (b), and multileaf collimator (MLC) (c), before (left bar) and after (right bar) registration

**FIGURE 3:**
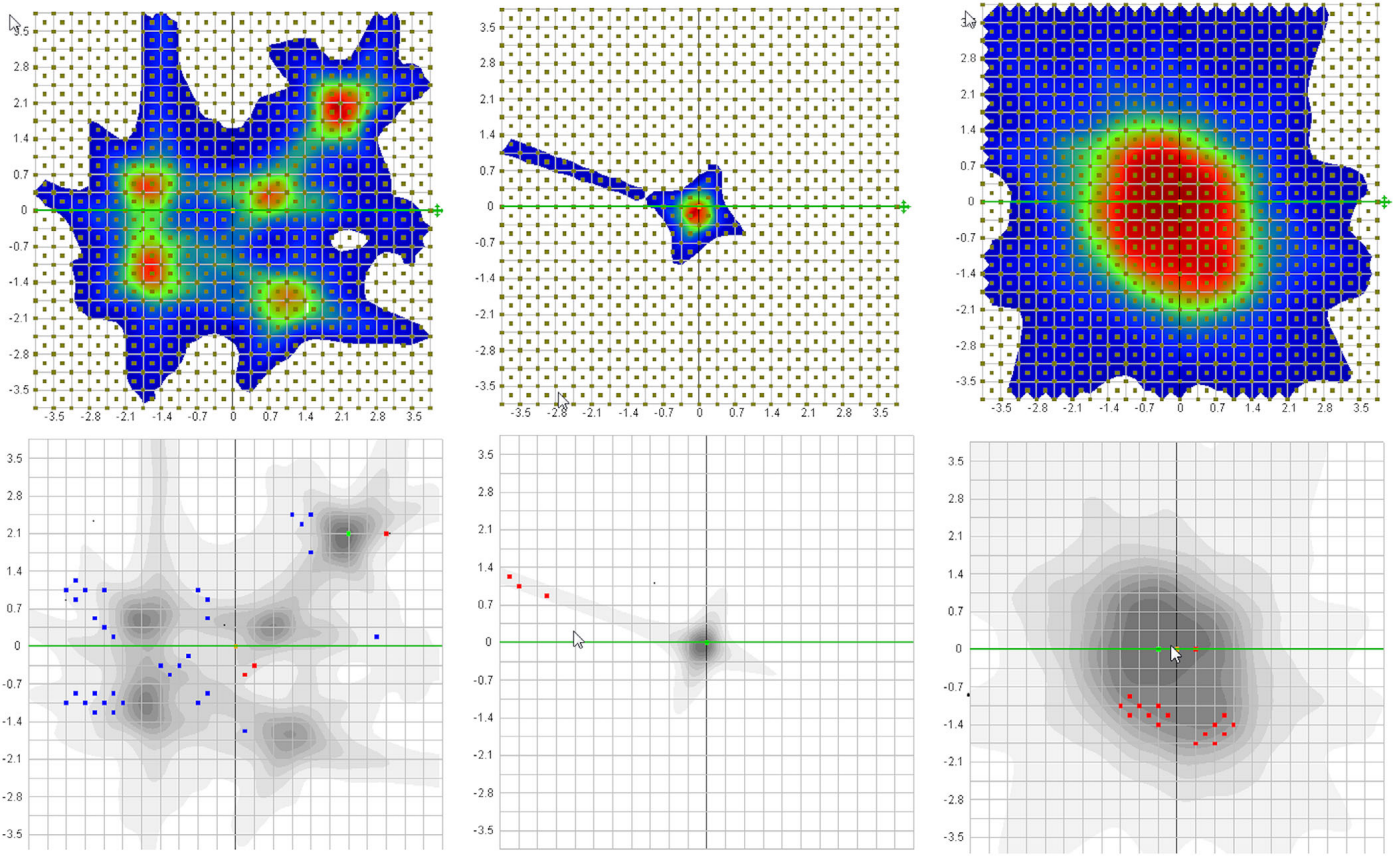
An example of the lowest passing rate in each category (before registration). The left one was the multiple metastases (METs) case with the lowest passing rate (94.4%, 3%/1 mm%) and most failed points were in the low‐dose areas between METs. The passing rate was improved to 99.8% after registration. The middle was a trigeminal neuralgia case with passing rate of 94.8% before registration and 98.2% after registration (1%/1 mm). The failed points were mainly in the low‐dose tail. The right one was a large tumor treated with multileaf collimator (MLC). The passing rate was 98.1% before registration and 99.8% after registration (3%/1 mm)

All the results were evaluated with the criterion of 90% passing rate for 0.5%–3% global dose‐difference, 1 mm distance‐to‐agreement, and 10% dose threshold. TG‐218 does not specify the gamma passing rate for SRS/SBRT cases but recommends tighter passing criterion and criterion that are appropriate given nearby organs at risk.^24^ The criterion chosen in the study was tighter than traditional intensity‐modulated radiation therapy as well as consistent with other studies.^6^, ^11^ The software also provided a unique shift/rotation registration algorithm to correct for potential setup errors between measurement and treatment planning systems (TPS) dose and we reported both results before and after registration. For criterion of 3%/1 mm, the passing rates were plotted in Figure 2a–c for the fixed cone, Iris, and MLC, respectively. The left bar was passing rate before registration and the right bar was after applying shifts in *x*, *y*, and *z* and angles (pitch (*α*), roll (*β*), and yaw (*γ*) in IEC coordinate system). Overall, the passing rates before registration for all the patients were above 90% for all three treatment modalities (96.8 ± 3.5%, the lowest passing rate of 90.4%), and were improved after registration (99.3 ± 1.5%). However, the passing rate varied significantly depending on the treatment sites and modalities. For single MET with fixed cone (patients 1–4 in Figure 2a), the passing rates were close before and after registration (99.1 ± 1.1% vs. 100 ± 0.0%). For multiple METs with fixed cone (patients 5–9 in Figure 2a), the passing rates were improved from 94.1 ± 2.1% to 99.3 ± 0.7% after registration. The relative lower passing rates before registration were due to the low‐dose bridges between METs. For TGN cases (patients 10–14 in Figure 2a), the passing rates were all 100% before applying registration. For Iris cases in Figure 2b, the passing rates before correction were the lowest (93.3 ± 3.4%) compared to the fixed cone and MLC and were improved to 98.2 ± 2.1% after registration. For MLC cases in Figure 2c, the passing rates were consistently high before correction (99.2 ± 0.8%) versus after correction (99.8 ± 0.4%). A few examples with the lowest passing rate before registration in each category (multiple METs, TGN, and large tumors) are shown in Figure 3.

Since the passing rates for all cases were at high 90 s after registration, we also analyzed the results with a tighter criterion (2%/1 mm). The post‐registration passing rates dropped from 99.3 ± 1.5% to 97.3 ± 3.2% with the tighter gamma criteria. Additionally, for TGN cases, we applied 0.5% and 1 mm criterion and the passing rates dropped from 100 ± 0.0% to 98.5 ± 2.0%. We also evaluated the impact of dose grid resolution on the passing rate by reducing the resolution. For CyberKnife high‐resolution plans, the dose resolution was the same as the planning CT (axial resolution from 0.68 to 0.98 mm depending on the size of axial field‐of‐view and slice thickness was 1.25 mm). When switched to medium resolution, the axial voxel size was doubled and the slice thickness was the same. The mean passing rates dropped 1.0% and 0.5% before and after registration when comparing to the high‐resolution plans for all the sites. The time for the entire QA process was another important factor for CyberKnife PSQA. Since phantom setup and data process were quick, plan delivery time accounted for most of the QA time and was recorded for each modality. The mean delivery time was 33.4 ± 11.7 min, 24.0 ± 4.9 min, and 17.1 ± 2.6 min for the fixed cone, Iris, and MLC, respectively. There were two options to further reduce delivery time: scaling MU and delivering beam in one nominal angle. None of these options were employed in this study.

## 4 DISCUSSION

In this study, we described implementation details of using SRS MapCHECK for PSQA for CyberKnife and retrospectively irradiated patient plans from various SRS sites and reported QA results from fixed cone, Iris, and MLC collimation types. We found reports on using SRS MapCHECK for CyberKnife were very rare and expected that our results could be used by other CyberKnife users for cross‐comparison. For linac SRS/SBRT PSQA, Rose et al.^6^ performed a multi‐institution validation study that collected PSQA results for 84 patient plans using SRS MapCHECK. For 3%/1 mm criterion, the mean gamma passing rate was 94.7% after registration and for 11% of the cases, the passing rates were below 90%. The main sources of error attributed to phantom setup, TPS dose grid spacing, TPS commissioning, plan modulation, etc. Our results were greatly improved and the mean passing rates were 96.8 ± 3.5% and 99.3 ± 1.5% before and after registration, respectively. None of the cases had passing rates below 90% before registration. We believe the major difference was the phantom setup and delivery accuracy. In Rose et al.,^6^ the phantom was aligned by either laser or cone beam computed tomography (CBCT) at different institutions. For one institution, they found that the imaging center of the CBCT was offset from the mechanical isocenter after investigation of a failed case. For CyberKnife, fiducial tracking was used for phantom setup and delivery, where four fiducials were placed around the detectors. Fiducials and detectors were integrated into one hardware, eliminating most setup uncertainties, for example, laser alignment with external marked lines on StereoPHAN, etc. Furthermore, our couch was a 6D robotic couch, which was capable of correction for pitch, roll, and yaw angles during setup. After finishing setup, there were always small residual shifts and rotation angles before delivery, which could be further corrected by the robot during delivery. Overall, CyberKnife fiducial tracking method has been proven to have submillimeter delivery accuracy, which was validated by our daily and monthly QA and other literature.^25^ We also evaluated the impact of dose grid resolution on the passing rate. After deceasing the resolution by doubling the dose grid size, the mean passing rates dopped by 1% before registration and 0.5% after registration. Since our delivery plans were always calculated with high resolution, the impact was likely minimal in our study.

**FIGURE 4:**
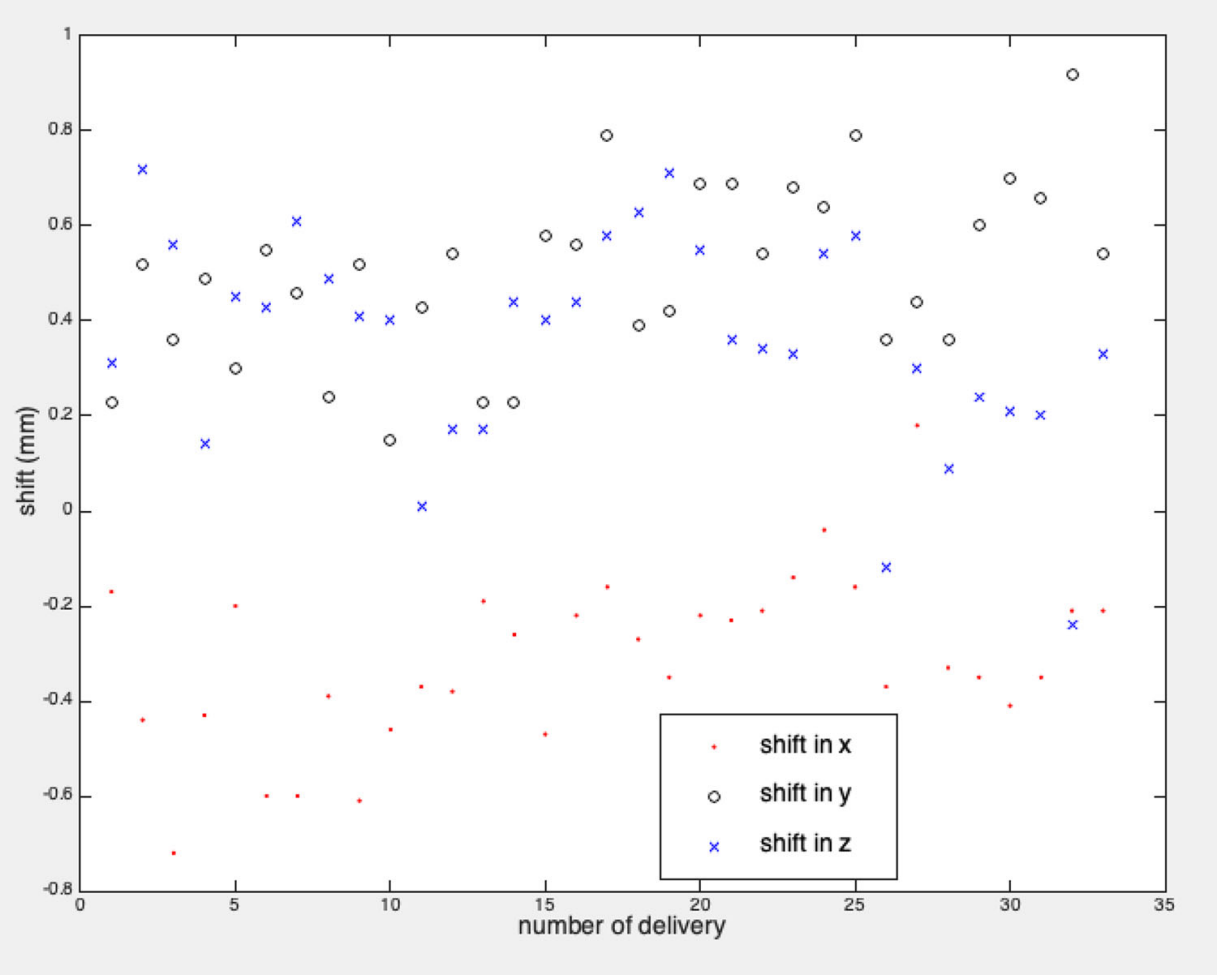
Shifts in *x*, *y*, and *z* for the cases after registration

We also explored potential sources of uncertainties in our study as there was 2.5% difference in the mean passing rates before and after registration. The resulting shifts in *x*, *y*, and *z* after registration for all treatment modalities were plotted in Figure 4. The mean of the shifts was ‐0.3 ± 0.2 mm, 0.5 ± 0.2 mm, and 0.4 ± 0.2 mm in *x*, *y*, and *z*, respectively. We noticed that the shift in each coordinate followed a consistent trend, even though the deliveries were done in a span of 2 months. Additionally, the shift variations were consistent between fixed cone, Iris, and MLC. We hypothesize that these small shifts could be due to inherent CyberKnife delivery inaccuracy, for example, uncertainties of the target locating system. Another source of the systematic error could be due to delineation error of the fiducials in the scanned phantom. Due to the high density of the fiducials, imaging artifacts were observed around each fiducial. Instead of using the automatic feature in Precision, we manually defined the fiducial in the CT by fine tuning its location. A small delineation error could introduce a systematic error in the QA results. The delineation accuracy could also relate to the CT slice thickness. The CT slice thickness in our study was 1.25 mm, which met the guidelines from TG‐101 and Medical Physics Practice Guideline 9a.^26^, ^27^ If it is decreased to 1 mm, the accuracy of fiducial delineation and dose calculation could be further improved. Also the position accuracy of diodes in the device could attribute to the overall uncertainties in the results. The laser accuracy could also contribute to the uncertainties in the array calibration, if laser was chosen to setup the phantom for array calibration.

One other source of uncertainty could be linac output variation during the day of delivery. For one patient QA, we observed relatively lower passing rate after delivery. When the software was switched to relative dose mode, the pass rate improved significantly. We checked the morning QA log and found the output in the morning was 2.2% higher. After adjusting the linac output, a second QA plan was delivered with high passing rate. We feel SRS MapCHECK could be one of the devices to check machine performance (e.g., beam profile, E2E, etc.) after linac major component replacement. As mentioned earlier, we used the array calibration factors provided by the vendor. This could lead to minor difference in the array calibration factors due to energy spectrum differences between machines. Initially we tried to measure the array calibration by ourselves based on laser alignment, but we noticed large geometric uncertainties were induced due to limited laser positioning accuracy and the FFF beam's gradient. Alternatively, this could be improved by designing delivery using fiducial tracking. Although eight treatment plans are needed, it may be worth a future investigation on the sensitivity of machine dependence for the array calibration.

## 5 CONCLUSIONS

In this study, a high‐resolution diode array was introduced and evaluated for PSQA of CyberKnife brain SRS/SRT. With superior gamma passing rates and reasonable QA time, we believe the SRS MapCHECK could be a good option for daily PSQA for CyberKnife SRS/SRT.

## CONFLICT OF INTEREST

The authors declare no conflict of interest.

## AUTHOR CONTRIBUTIONS

Qianyi Xu and Jiajin Fan contributed to the conception, design, and analysis of the study. The first draft of the manuscript was written by Qianyi Xu and the rest of authors commented and edited the manuscript. We confirm that all coauthors contributed to the study.
